# Patterns of Plant Biomass Allocation in Temperate Grasslands across a 2500-km Transect in Northern China

**DOI:** 10.1371/journal.pone.0071749

**Published:** 2013-08-20

**Authors:** Wentao Luo, Yong Jiang, Xiaotao Lü, Xue Wang, Mai-He Li, Edith Bai, Xingguo Han, Zhuwen Xu

**Affiliations:** 1 State Key Laboratory of Forest and Soil Ecology, Institute of Applied Ecology, Chinese Academy of Sciences, Shenyang, China; 2 Forest dynamics, Swiss Federal Research Institute WSL, Birmensdorf, Switzerland; 3 State Key Laboratory of Vegetation and Environmental Change, Institute of Botany, Chinese Academy of Sciences, Beijing, China; 4 Graduate University of Chinese Academy of Sciences, Beijing, China; DOE Pacific Northwest National Laboratory, United States of America

## Abstract

Plant biomass allocation between below- and above-ground parts can actively adapt to the ambient growth conditions and is a key parameter for estimating terrestrial ecosystem carbon (C) stocks. To investigate how climatic variations affect patterns of plant biomass allocation, we sampled 548 plants belonging to four dominant genera (*Stipa spp.*, *Cleistogenes spp.*, *Agropyron spp.*, and *Leymus spp.*) along a large-scale (2500 km) climatic gradient across the temperate grasslands from west to east in northern China. Our results showed that *Leymus spp.* had the lowest root/shoot ratios among the each genus. Root/shoot ratios of each genera were positively correlated with mean annual temperature (MAT), and negatively correlated with mean annual precipitation (MAP) across the transect. Temperature contributed more to the variation of root/shoot ratios than precipitation for *Cleistogenes spp*. (C4 plants), whereas precipitation exerted a stronger influence than temperature on their variations for the other three genera (C3 plants). From east to west, investment of C into the belowground parts increased as precipitation decreased while temperature increased. Such changes in biomass allocation patterns in response to climatic factors may alter the competition regimes among co-existing plants, resulting in changes in community composition, structure and ecosystem functions. Our results suggested that future climate change would have great impact on C allocation and storage, as well as C turnover in the grassland ecosystems in northern China.

## Introduction

Below-ground carbon (C) is the primary component of C stocks in grassland ecosystems, accounting for >80% of plant C stocks [1]–[3]. The below-ground system plays an important role in controlling terrestrial C sequestration and cycling. However, compared to the above-ground system, knowledge of the below-ground system is still limited [4]. Root/shoot ratios have been used to calibrate and estimate C storage from the more easily measurable aboveground biomass [5]. As a critical determinant, root/shoot ratios have also been incorporated into terrestrial ecosystem C modeling [6]–[7]. Quantifying root/shoot ratio and its relationships with climatic factors is not only important for improving our understanding of biomass allocation in terrestrial ecosystems, but also critical for predicting global C sequestration and cycling under climate change. To our knowledge, however, few studies have investigated the influences of variations in climate on root/shoot ratios across geographical scales [8].

From a physiological perspective, root/shoot ratio is usually thought to reflect the differential investment of photosynthates between above- and below-ground organs, induced by abiotic and biotic pressures [4], [9]–[10]. For example, plants would allocate relatively more biomass to roots if their growth is more strongly influenced by below-ground factors (e.g. nutrients, water), whereas they would allocate relatively more biomass to shoots if their growth is more strongly influenced by above-ground factors (e.g. light, CO_2_) [11]–[12]. Both temperature and water availability are important abiotic factors affecting plant growth [8] and plant biomass allocation [13]–[15]. In previous studies, plant root/shoot ratio in grassland was found to significantly decrease with increasing mean annual temperature and precipitation [4], [16]. However, other studies did not find changes of plant root/shoot ratios with the variation of temperature and precipitation at both individual [17] and community levels [18] in China’s grasslands. The inconsistency may be due to different plant types investigated. Different plant genera, plant functional groups (e.g. C3 and C4 plants), and plant growth groups (e.g. isometric and allometric plants) may have different specialized strategies to regulate their root/shoot ratios in order to maximize the use of external resources [19]–[23]. Therefore, it is important to investigate how different plant genera respond to the changing climatic factors.

Temperate grassland is one of the most widespread biomes on earth and plays a key role in global C cycling [24]. The temperate grassland in northern China, located in the eastern part of the Eurasian grassland biome, supports diverse species of plants and animals and serves the development of socio-economics of the region [25]. Additionally, this grassland, located in temperate continental arid region, has a remarkable transition of climate, soil, vegetation, and biogeochemical cycling from west to east [26]–[28], which offers a unique opportunity for examining the characteristics of grasslands in relation to climatic factors.

Here, we investigated plant biomass allocation between above- and below-ground in four dominant genera (*Stipa*, *Cleistogenes*, *Leymus* and *Agropyron*) along a large-scale transect of approximately 2500 km across the temperate grasslands of northern China. Plants of these four genera provide the majority of the community primary production, and to a great extent, determine the structure and function of the grassland ecosystem. We aimed to: (1) document the general patterns of root/shoot ratios of these four genera across geographical scale, (2) examine the effects of climatic factors on their root/shoot ratios, and (3) illustrate the relative contribution of different climatic variables to the variation of these root/shoot ratios.

## Materials and Methods

### Study Area and Sampling Sites

**Figure 1:**
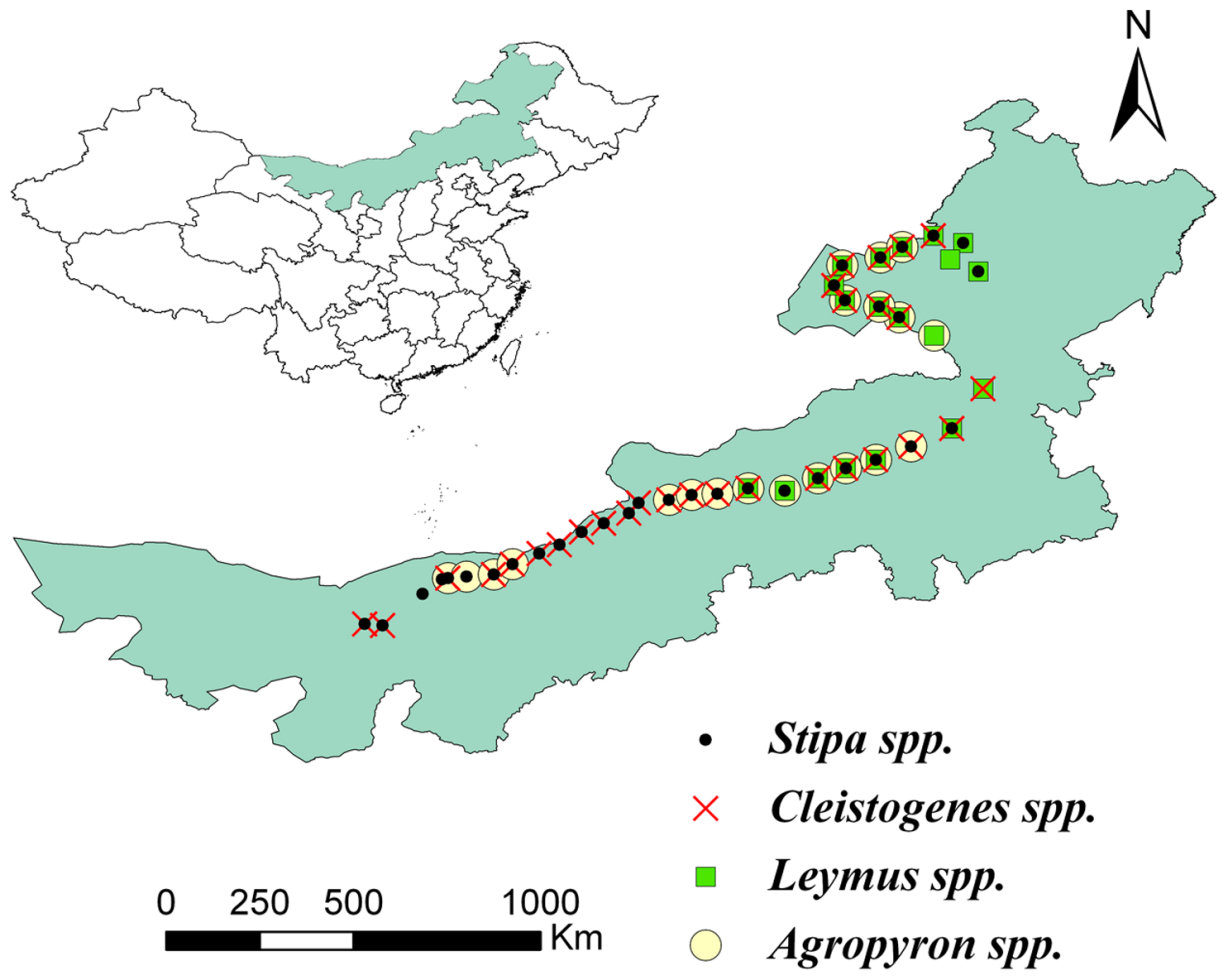
Locations of the sampling sites along the transect across the temperate grasslands of northern China.

The research was conducted in the semi-arid region of northern China. The selection of sample sites was principally defined by a grassland transect of ∼2500 km ranging from west to east across the temperate grassland of northern China (Figure 1). The longitudinal-latitudinal transect covers latitudes from 40.7°N to 50.1°N and longitudes from 105.6°E to 120.4°E (Figure 1). Across the broad geographical regions and environmental gradients along the west-east transect, there are various vegetation types such as the desert grassland in the western part, the typical grassland in the middle part, and the meadow grassland predominantly in the eastern part.

The investigation was conducted in August 2012 when the phenological difference was relatively negligible and the grasses had matured across the transect. All necessary permits were gained before the beginning of field investigation. Sample sites subjected to minimal grazing and other anthropogenic disturbances were visually selected in the field. Exact sampling locations were GPS-referenced with latitude, longitude and elevation (eTrex Venture, Garmin, ±3 m accuracy). A total of 37 sample sites were investigated along the 2500 km grassland transect and the distance between two adjacent sample sites was about 60 km.

### Plant Materials

*Stipa*, *Cleistogenes*, *Leymus,* and *Agropyron* are four most widely distributed dominant genera in the temperate grasslands in northern China. Dominant plants belonging to these four genera along the transect were sampled. Both *Stipa spp.* and *Agropyron spp.* are perennial multi-stems C3 bunchgrasses, *Leymus spp.* are perennial single-stem C3 rhizome grasses, and *Cleistogenes spp.* are multi-stems C4 bunchgrasses [29].

### Sampling

At each sampling site, ten sampling plots (1-m×1-m each) were established along a 500-m long sampling strip which crosses the transect perpendicularly. The distance between two adjacent plots was 50 m. Five to ten mature and healthy individuals belonging to each of the four genera (*Stipa*, *Cleistogenes*, *Agropyron,* and *Leymus*) were randomly sampled if existed (Figure 1). The maximum plant height of four dominant genera was measured with a ruler. The majority of root material of these plants were located in the upper 30-cm of soil [29]. The root system was excavated by extracting a soil cylinder of about 15–25 cm in diameter and 20–30 cm in depth with the target plant in the middle, using a spade. The size of the soil cylinder depended on the aboveground morphology of the target plant. Roots of the target plant were carefully collected and separated from soils and other belowground materials. The roots and shoots were then separated from the ground surface [4] and put into paper bags separately. Afterwards, the roots were washed free of soils under running water for 5 minutes before all plant samples were oven-dried at 80°C to constant weight. Root/shoot ratio is defined as the dry root biomass (g) divided by the dry shoot biomass (g) (Appendix S1). Since these data were all collected by a single team of researchers using uniform collection method, this analysis avoids the difficulty of heterogeneous data which previous large-scale analyses of biomass allocation have encountered.

### Climate Data

Five climatic variables, i.e. mean annual temperature (MAT, °C), mean annual precipitation (MAP, mm), mean growing season temperature (GST, °C), mean growing season precipitation (GSP, mm) and mean annual potential evapotranspiration (PET, mm year^−1^), were employed to analyze the climatic controls on the spatial patterns of plant root/shoot ratios. Monthly mean temperature and monthly mean precipitation were extracted from a global climate dataset with a resolution of 0.0083°×0.0083° (approximately 1 km^2^ at the equator) obtained from http://www.worldclim.org/. Growing season was defined as the months with a mean temperature ≥5°C. PET was extracted from Numerical Terradynamic Simulation Group with a resolution of 0.5°×0.5° obtained from http://www.ntsg.umt.edu/project/mod16. A drought index (DI) based on cumulative values of precipitation and potential evapotranspiration for each site across the transect was calculated, using MAP/PET (Appendix S1).

### Statistical Analysis

The Kolmogorov-Smirnov (KS) test was used to test for normality of data before statistical analysis [30]. Differences among means were compared by one-way ANOVA, followed by multiple comparison tests. Semi-variogram analysis was employed to check the spatial autocorrelation of our sampling sites [31]. Results showed that the range of the spatial dependency of MAT and MAP (25 km and 22 km, respectively) were lower than the distance between two adjacent sample sites (60 km), which indicated our sample points had non-significant spatial autocorrelation (Table 1).

**Table 1:** Semi-variogram test of spatial autocorrelation of mean annual temperature (MAT) and mean annual precipitation (MAP) among sampling sites across a 2500-km long transect in northern China.

	Model	Nugget effect	Sill	Range	R^2^
MAP	Gaussian	1030	15280	25	0.945
MAT	Gaussian	0.3	7.6	22	0.905

Relationships between root/shoot ratios and climatic factors were examined with linear mixed model incorporating random effects, using sample plots as the random factor and climatic variables as the fixed factors [32]. All statistical analyses were performed using the R software package (version 2.15.0). To test the independent contribution of temperature and precipitation to root/shoot ratios, we conducted path analysis using SAS 9.0 (SAS Institute Inc., Cary, NC, USA). Path analysis can be used to partition the relationship between root/shoot ratios and climatic factors into direct and indirect effect [33]. A high path coefficient indicates a strong contribution of a climatic factor (MAP, MAT, GSP, and GST) to root/shoot ratios. Direct path coefficients measure direct effects of a climatic factor (MAP, MAT, GSP, and GST) on root/shoot ratios, while indirect path coefficients specify the effects of a climatic factor passed through other climate factors. Path analysis cannot exclude the random effects of sample plots. Hence, we used the mean root/shoot ratio of each site to demonstrate the independent contribution of temperature and precipitation to root/shoot ratios. This paper only presents the results of MAP and MAT, since the effects of GSP and GST were not different from those of MAP and MAT.

Allometric relationships between traits have been generally understood as exponential relationships described by Log y = a (Log x)+b, where “x” and “y” are the two variables, “a” represents the scaling slope and “b” the intercept [34]. The relationship between the log-transformed root biomass and shoot biomass was tested with standardized major axis (SMA) regression. SMA regression protocols are appropriate when both regression variables are interdependent and subject to measurement errors as in the case of shoot biomass and root biomass [35]. SMA slopes and intercepts were obtained using the SMART software package developed by Falster et al. [36]. SMA slopes were tested against 1.0. Non-significant difference from 1.0 in the slopes indicates an allometric relationship between root and shoot biomass [35].

## Results

### Root/Shoot Ratios of the Four Dominant Genera

**Figure 2:**
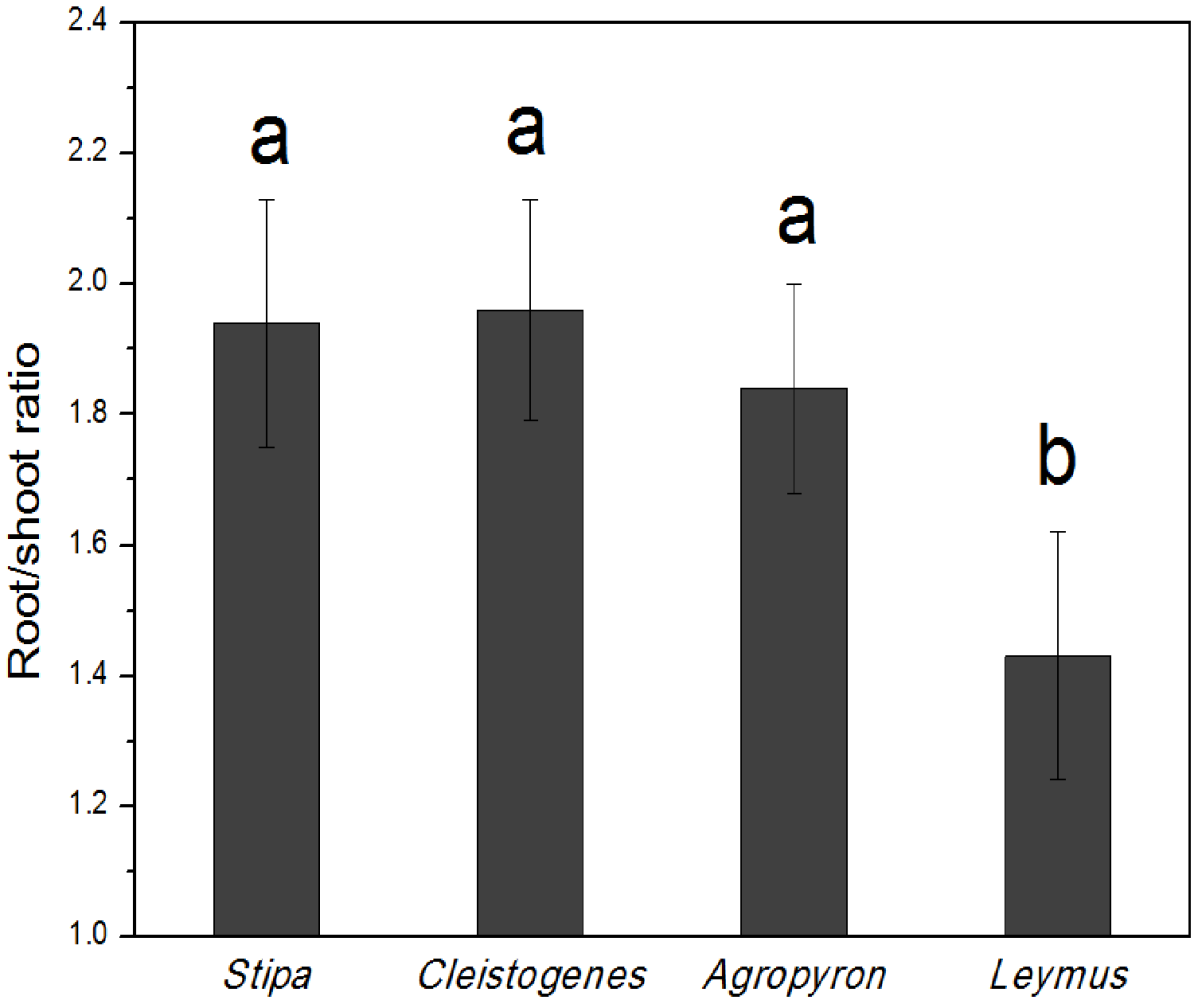
Root/shoot ratios (mean ±1 SE) of four grasses genera (*Stipa*, *Cleistogenes*, *Agropyron*, and *Leymus*). Bars with dissimilar letters denote signiffcant difference (*p*<0.01).

Plant root/shoot ratios were significantly drawn from a normally distributed population for each genus (Table 2). Mean root/shoot ratio was 1.94 (ranging from 1.12 to 2.72) for *Stipa spp.*, 1.97 (1.15–2.98) for *Cleistogenes spp.*, 1.84 (1.22–2.50) for *Agropyron spp.*, and 1.43 (0.75–2.4) for *Leymus spp.* (Table 2; Figure 2), whereas the mean maximum plant height was 19 cm (ranging from 5 to 49 cm) for *Stipa spp.*, 10 cm (3–23 cm) for *Cleistogenes spp.*,19 (10–25 cm) for *Agropyron spp.* and 32 cm (13–54 cm) for *Leymus spp.* (Table 2). CV of root/shoot ratios ranged from 13% to 21% (Table 2).

**Table 2:** Statistics of plant root/shoot ratios of four dominant genera (*Stipa*, *Cleistogenes*, *Agropyron*, and *Leymus*) across a 2500-km long transect in northern China’s temperature grassland.

Genus	n	Range	Mean ± SD	CV (%)	K-S test	MPH(cm)
*Stipa*	196	1.12–2.72	1.94±0.29	14.95	Normal	19(5–49)
*Cleistogenes*	151	1.15–2.98	1.97±0.27	13.71	Normal	10(3–23)
*Agropyron*	96	1.22–2.50	1.84±0.26	14.13	Normal	19(10–25)
*Leymus*	105	0.75–2.40	1.43±0.29	20.28	Normal	32(13–45)

Number of replicates (n), mean, range, standard deviation (SD), coefficient of variation (CV: defined as SD/mean) and maximum plant height (MPH) and Kolmogorov-Smirnov (K-S) test results are all reported.

### Relationships between Root/Shoot Ratios and Climatic Factors

Linear mixed model analysis showed that root/shoot ratios were positively correlated with MAT (all *p*<0.05) and negatively correlated with MAP (all *p*<0.001) excluding the random effects of sample plots at both intra- and inter-genus levels (Table 3). Path analysis showed that direct dependence of root/shoot ratios on MAP were greater than on MAT for *Stipa spp.*, *Agropyron spp.* and Leymus *spp.* (Table 4). For *Cleistogenes spp.*, however, MAT had stronger direct effects on root/shoot ratios than MAP (Table 4). The direct path coefficient of MAP on the root/shoot ratios was −0.577 for *Stipa spp.*, −0.231 for *Agropyron spp.*, −0.417 for *Leymus spp.* and −0.413 for *Cleistogenes spp.*, with the indirect path coefficient via MAT of −0.252, −0.399, −0.053 and −0.064, respectively. The direct path coefficient of MAT were 0.285 for *Stiap spp.*, 0.468 for *Cleistogenes spp.*, 0.064 for *Agropyron spp.,* and 0.21 for *Leymus spp.* with the indirect path coefficient via MAP of 0.509, 0.21, 0.342 and 0.126, respectively.

**Table 3:** Effects of MAT and MAP on root/shoot ratios of four dominant genera (*Stipa, Cleistogenes, Agropyron*, and *Leymus*) across a 2500-km long transect in northern China’s temperature grassland.

Root/shoot ratiosfor *Stipa*				
Fixed effect				
Variables	Slope	Intercept	R	*p*-value
MAT	0.071	1.77	0.629	*p*<0.001
MAP	−0.002	2.33	0.938	*p*<0.001
Root/shoot ratios for *Cleistogenes*				
Fixed effect				
MAT	0.054	1.85	0.646	*p*<0.001
MAP	−0.001	2.30	0.936	*p*<0.001
Root/shoot ratiosfor *Agropyron*				
Fixed effect				
MAT	0.040	1.80	0.468	*p*<0.05
MAP	−0.001	2.24	0.972	*p*<0.001
Root/shoot ratiosfor *Leymus*				
Fixed effect				
MAT	0.059	1.46	0.269	*p*<0.05
MAP	−0.002	2.06	0.986	*p*<0.001
Root/shoot ratios forall four genera				
Fixed effect				
MAT	0.076	1.72	0.266	*p*<0.05
MAP	−0.001	2.14	0.824	*p*<0.001

MAT, mean annual temperature; MAP, mean annual precipitation.

A linear mixed model was employed, using sample plots as the random factor and climate variables as the fixed factors. R is the correlation coefficients between climate factors and root/shoot ratios. P-values are estimated using restricted maximum likelihood (REML) estimates and are reported for significant (*p*<0.05) model terms.

**Table 4:** Path coefficients between climate factors (MAT and MAP) and plant root/shoot ratios of four dominant genera (*Stipa*, *Cleistogenes*, *Agropyron*, and *Leymus*) across a 2500-km long transect in northern China’s temperature grassland.

Genus		R	Direct path	Indirect path coefficient	
			coefficient	Via MAP	Via MAT
*Stipa*	MAP–R/S	−0.829	−0.577		−0.252
	MAT–R/S	0.794	0.285	0.509	
*Cleistogenes*	MAP–R/S	−0.630	−0.231		−0.399
	MAT–R/S	0.678	0.468	0.210	
*Agropyron*	MAP–R/S	−0.470	−0.417		−0.053
	MAT–R/S	0.406	0.064	0.342	
*Leymus*	MAP–R/S	−0.477	−0.413		−0.064
	MAT–R/S	0.336	0.210	0.126	

MAT, mean annual temperature; MAP, mean annual precipitation; R/S, root/shoot ratio.

Indirect path coefficient means the indirect dependency of root/shoot ratios on certain climate factor and R is the correlation coefficient between the climate factors and the root/shoot ratios.

### Allometric Relationships between Root and Shoot Biomass

**Figure 3:**
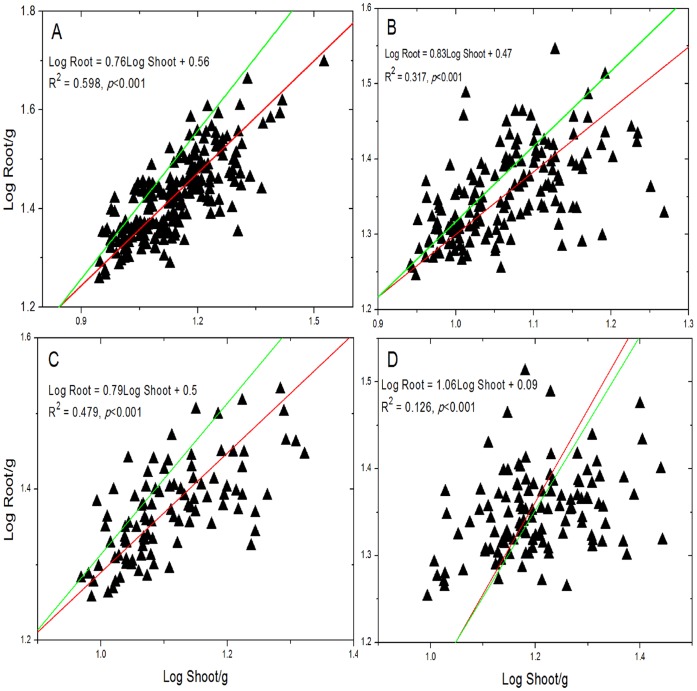
Allometric relationship between root biomass and shoot biomass. *Stipa spp.* (A), *Cleistogenes spp.* (B), *Agropyron spp.* (C), and *Leymus spp.* (D). Red lines are the standardized major axis regression curves (for a summary of regression statistics, see Table 5). Green lines are isometric lines with slope equal to 1 and y-intercept equal to that of the corresponding red lines.

Shoot biomass was positively correlated with root biomass (*p*<0.001) for each genus we sampled (Figure 3; Table 5). The allometric slope of the relationship between log-root and log-shoot biomass obtained by the reduced major axis analysis for *Stipa spp.*, *Cleistogenes spp.*, *Agropyron spp.* and *Leymus spp.* was 0.76 (with a 95% confidence interval of 0.69–0.83), 0.83 (0.72–0.94), 0.79 (0.68–0.91), and 0.93 (0.78–1.09), respectively (Table 5). These values differed significantly from 1.0, except for the last value (*p* = 0.360), indicating isometric growth for *Leymus spp.* and allometric growth for the rest three genera (Table 5).

**Table 5:** Linear regression between above- and below-ground biomass for *Stipa spp.*, *Cleistogenes spp.*, *Agropyron spp.*, and *Leymus spp.* using a standardized major axis (SMA) method.

Genus	R^2^	Slope(95% CI)	Intercept	*p* (H_0_:slope = 1.0)
*Stipa*	0.60***	0.76 (0.69−0.83)	0.56 (0.48−0.63)	<0.001
*Cleistogenes*	0.32***	0.83 (0.72−0.94)	0.47 (0.36−0.59)	<0.001
*Agropyron*	0.48***	0.79 (0.68−0.91)	0.50 (0.37−0.63)	<0.01
*Leymus*	0.13***	1.06 (0.88−1.16)	0.09 (−0.21-0.06)	= 0.360

95% CI, 95% confidence interval; ***, *p*<0.001.

Both above- and below-ground biomass were log10–transformed. *p* is the significance level at which the estimated slopes are different from 1.0.

## Discussion

Our results showed that *Leymus spp.* had significantly lower root/shoot ratios than *Stipa spp.*, *Cleistogenes spp.* and *Agropyron spp.* (Figure 2). This could be partly explained by the relatively high plant height of *Leymus spp.*, since root/shoot ratios generally deceased with increasing plant height [37]. Li et al. [37] found that root/shoot ratios of grasses decreased logarithmically with increasing plant height in the temperate grasslands of northern China. Chapin and Chapin [38], Snowdon et al. [39], and Henry and Thomas [40] also reported that shorter plants usually had higher root/shoot ratios in natural grasslands, shrubs and forests ecosystems, respectively.

Root/shoot ratio is considered to be a strong indicator for the capacity of plants to take up light, water and nutrients. Plants would demonstrate optimal distribution patterns to adapt to particular environmental conditions, such as shade or drought [48]–[49]. Grasses grown in drought environment are expected to allocate more biomass to root systems for water uptake [41], [50]. Our data within each genus and across the four genera supported this hypothesis. From west to east of our sampling transect, MAP increased from 100 mm to 450 mm and MAT decreased from 8.1°C to −3.1°C. Along the transect, plants allocated, correspondingly, less biomass to root system for taking up soil water and more biomass to shoot system for light, leading to a decreasing trend in root/shoot ratios from west to east. Warming may stimulate evaportranspiration and reduce soil water content, which explains the observed higher root/shoot ratios with increasing MAT. Moreover, lower temperature in the eastern part of the transect may have more negatively influenced the root growth rather than the shoot growth. This is consistent with what Kummerow [42] found in arctic sedges, where lower root/shoot ratios were observed in areas with lower temperature, indicating that a relatively large fraction of the photosynthate was allocated to shoot production under cold conditions. Mokany et al. [4] found that root/shoot ratios in grassland decreased significantly with both increasing MAP and increasing MAT (*p*<0.001). Yang et al. [18] found that root/shoot ratios did not vary markedly with MAP and MAT for grassland plants at the community level in China. However, our results indicated positive relationships between root/shoot ratios and MAT but negative relationships between root/shoot ratios and MAP for the four dominant genera across the west-east transect (Table 3), suggesting positive effects of temperature or radiation but negative effects of water availability on root/shoot ratios in arid and semi-arid regions. Our results agreed with findings of Xu et al. [43], which indicated that high temperature in combination with soil drought increased the root/shoot ratios of *Leymus chinensis*. Temperature has been well known for stimulating soil enzyme activities and increasing decomposition and mineralization of organic matter [44]. Additionally, temperature can also stimulate nutrient transportation in soils and nutrient uptake by roots [45]. Hence, plant root/shoot ratios are expected to decrease with increasing temperature under favorable below-ground conditions.

However, in arid and semi-arid regions, water stress becomes the most limiting factor for plant growth. Therefore, the influence of temperature on C allocation is mainly reflected in its interaction with the limited precipitation [22], [35], [46], explaining the positive correlation between temperature and root/shoot ratios we found. Our results may also be partly explained further by the significantly positive correlation between root/shoot ratios and PET (all *p*<0.05) and the significantly negative correlation between root/shoot ratios and DI (all *p*<0.05) (Table 6). Our results suggested that future climate change characterized by increases in both temperature and precipitation in the semiarid grasslands of northern China may result in changes in C allocation between the above- and belowground part of plants in this ecosystem.

**Table 6:** Effects of PEI and DI on root/shoot ratios of four dominant genera (*Stipa*, *Cleistogenes*, *Agropyron*, and *Leymus)* across a 2500-km long transect in northern China’s temperature grassland.

Root/shoot ratiosfor *Stipa*				
Fixed effect				
Variables	Slope	Intercept	R	*p*-value
PET	0.001	0.47	0.992	*p*<0.001
DI	−0.099	2.26	0.903	*p*<0.001
Root/shoot ratiosfor *Cleistogenes*				
Fixed effect				
PET	0.001	0.97	0.992	*p*<0.001
DI	−1.747	2.25	0.970	*p*<0.001
Root/shoot ratiosfor *Agropyron*				
Fixed effect				
PET	0.001	1.32	0.993	*p*<0.05
DI	−1.530	2.12	0.952	*p*<0.05
Root/shoot ratiosfor *Leymus*				
Fixed effect				
PET	0.001	0.91	0.996	*p*<0.05
DI	−2.201	1.97	0.980	*p*<0.05
Root/shoot ratios forfour genera				
Fixed effect				
PET	0.001	0.33	0.991	*p*<0.001
DI	−2.687	2.32	0.911	*p*<0.001

PEI, mean annual potential evapotranspiration; DI, drought index.

A linear mixed model was employed, using sample plots as the random factor and climate variables as the fixed factors. R is the correlation coefficients between climate factors and root/shoot ratios. P-values are estimated using restricted maximum likelihood (REML) estimates and are reported for significant (*p*<0.05) model terms.

According to theoretical predictions and prior observations, plant growth strategies should be mainly regulated by precipitation in the arid and semi-arid grassland of northern China because water availability is the dominant limiting factor for primary productivity in this region [46]. However, our results revealed that the relative contribution of MAT and MAP to the variations of root/shoot ratios was genus-dependent. Root/shoot ratios of C3 plants (*Stipa spp.*, *Agropyron spp.* and *Leymus spp.*) were more influenced by precipitation, whereas root/shoot ratios of C4 plants (*Cleistogenes spp.*) were more controlled by temperature. James et al. [47] and Wang [48] also found similar phenomena. This can be explained by the more effective quantum yield of CO_2_ fixation [49] and the higher maximum photosynthetic rate of C4 plants at higher temperature [50]. Therefore, more C can be invested to roots for nutrients uptake, resulting in higher root/shoot ratios at higher temperature. In addition, C4 pathway could overcome the deleterious effects of drought on photosynthesis and enables C4 plants to photosynthesize more efficiently at lower water content and higher temperature. C4 plants usually have higher photosynthetic water-use efficiency than C3 plants [51]. Similarly, Pau et al. [52] demonstrated that C4 grasses showed a preference for regions with higher temperatures and lower precipitation compared with C3 grasses at the global scale. Sage and Kubien [50] suggested that C4 species may be more readily respond to warming in a manner that promotes rapid root proliferation and colonization in disturbed habitats. This is consistent with the widely held but unproven hypotheses that C4 plants should be favored by global warming [49].

The isometric hypothesis suggests that above-ground biomass scales one-to-one with respect to the below-ground biomass at both individual and community levels [53], [54]. We only found that the scaling of root biomass vs. shoot biomass of *Leymus spp*. followed 1∶1 line (isometric plants) (Figure 3; Table 5). The other three genera (*Stipa*, *Cleistogenes* and *Agropyron*) were allometric plants, which showed asymmetrical change between root and shoot biomass with changing environments (Table 5; Figure 3). This difference among the four genera may be caused by the differences in plant morphology and may also be influenced by natural selection. It makes larger ecological sense that plants allocate more of their biomass to belowground in response to water limitation regardless of an isometric or allometric relationship between shoot and root biomass (Table 3; Table 6). Such an allometic or isometric relationship is controlled by both the slope (relative growth rate of shoot and root biomass) and the y-intercept (the absolute value of root/shoot ratio). All the y-intercepts for the four genera were significantly different from zero, indicating that the absolute values of root/shoot ratios for the four genera were not consistent. Therefore, our findings indicated that the relationship between root and shoot biomass in relation to environment conditions may be genus-specific.

### Conclusion

Our results suggested that plants in the temperate grassland would alter their carbon allocation pattern between above- and belowground parts in response to climate changes. Under warming condition, plants, especially C4 plants, may allocate relatively less biomass to aboveground, resulting in higher competitive capacity of C4 plants compared to C3 plants, which may finally change community structure and ecosystem function. Given the sensitivity of root/shoot ratios of the examined dominant genera to climatic factors, future climate change will have great impacts on C storage and turnover in the grasslands of northern China.

## Supporting Information

Appendix S1 **Dataset on the root/ratios of four dominant genera (** ***Stipa*** **,** ***Cleistogenes*** **,** ***Agropyron*** **, and** ***Leymus*** **), and information on the geographic location and climate of the study area.**
(XLSX)
